# First Complete Genome Sequence of a Lineage III Peste des Petits Ruminants Virus

**DOI:** 10.1128/genomeA.01054-14

**Published:** 2014-10-23

**Authors:** William G. Dundon, Simon M. Kihu, T. Bharani K. Settypalli, George C. Gitao, Lily C. Bebora, Njenga M. John, Julius O. Oyugi, Roland Silber, Angelika Loitsch, Adama Diallo

**Affiliations:** a Joint FAO/IAEA Division of Nuclear Techniques in Food and Agriculture, Department of Nuclear Sciences and Applications, International Atomic Energy Agency, Vienna, Austria; b Department of Veterinary Pathology, Microbiology and Parasitology, Faculty of Veterinary Medicine, University of Nairobi, Uthiru, Kenya; c University of Nairobi, Institute of Tropical and Infectious Diseases (UNITID), Nairobi, Kenya; d Institute for Veterinary Disease Control, Austrian Agency for Health and Food Safety, Moedling, Austria

## Abstract

We report the first complete genome sequence of a lineage III peste des petits ruminants virus (KN5/2011) using RNA extracted from goat lung tissue collected in Kenya in 2011. The genome shows the highest nucleotide sequence identity with lineage II peste des petits ruminants viruses (PPRVs) (86.1 to 87.2%) and the lowest with lineage IV PPRVs (82.5 to 83.8%).

## GENOME ANNOUNCEMENT

Peste des petits ruminants (PPR) is a highly infectious transboundary viral animal disease that affects mainly sheep, goats, and small wild ruminants. Sheep and goats contribute considerably to the cash income and nutrition of small farmers in many countries so the control of PPR, with morbidity and mortality rates of 70 to 80%, is considered an essential element in the fight for global food security and poverty alleviation (1).

The peste des petits ruminants virus (PPRV) is a member of the genus *Morbillivirus* within the family *Paramyxoviridae* (1). It is a nonsegmented, negative, single-stranded RNA virus that encodes six structural proteins; nucleocapsid protein (N), phosphoprotein (P), matrix protein (M), fusion protein, (F), hemagglutinin protein (H), RNA-dependent RNA polymerase (L), and two non-structural proteins (V and C). PPRVs have been classified into four genetic lineages based on the comparison of a sequence fragment of the N and/or F genes (2). Lineage IV is prevalent in Asian countries, while all four lineages have been found in Africa (1). To date, only eleven full genome sequences are available in public databases representing three of the four lineages.

In May 2011, in Turkana County, Kenya, tissue samples were collected from three goats suspected of dying of PPRV. The tissue samples were transported on ice to the University of Nairobi, stored at −80°C, and were shipped in May 2014 to the Austrian Agency for Health and Food Safety for further characterization. RNA was extracted from the samples and analyzed by reverse transcription (RT)-PCR to test for the presence of PPRV RNA as described previously (2). Phylogenetic analysis of the amplicons generated from positive tissue samples revealed that they contained viral RNA from a lineage III PPRV. The RNA from one positive lung sample was then selected for genome sequencing as described previously (3).

The organization of the KN5/2011 genome (15,948 bp) was as expected with a 107-nucleotide genome promoter region at the 3′ end followed by the transcription units for the N, P, M, F, H, and L proteins and the antigenome promoter at the 5′ end. The genome has the highest nucleotide sequence identity (87.2%) with the lineage II virus Nigeria 76/1 (EU267274) and the lowest identity (82.5%) with the lineage IV virus Sungri/96 (KF727981). It shares 84.7% nucleotide sequence identity with ICV89 (EU267273), the only full genome sequence of a lineage I PPRV available in GenBank. M is the most conserved of the proteins and has between 96.7 and 97.9% identity with lineage II viruses while the V protein is the least conserved having its highest identity (81.2%) with the lineage II viruses Nigeria 76/1 (EU267274) and Nigeria 75/1 (HQ197753).

This is the first available complete genome of a lineage III PPRV and provides important information that, in combination with data on experimental and field infections in animals, will provide a clearer understanding of the genetic influences on host specificity, viral pathogenicity, and transmission of PPRV.

### Nucleotide sequence accession number.

The complete genome sequence of KN5/2011 has been deposited in GenBank under accession no. KM463083.
